# Text Analysis of Electronic Medical Records to Predict Seclusion in Psychiatric Wards: Proof of Concept

**DOI:** 10.3389/fpsyt.2019.00188

**Published:** 2019-04-11

**Authors:** Mirjam C. Hazewinkel, Remco F. P. de Winter, Roel W. van Est, Dirk van Hyfte, Danny Wijnschenk, Narda Miedema, Erik Hoencamp

**Affiliations:** ^1^Clinical Centre for Acute Psychiatry, Parnassia, Parnassia Group, The Hague, Netherlands; ^2^Department of Clinical Psychology, VU University, Amsterdam, Netherlands; ^3^Data Research Office, Antes, Parnassia Group, Rotterdam, Netherlands; ^4^InterSystems BV Benelux, Vilvoorde, Belgium; ^5^Department of Clinical Psychology, Institute of Psychology, Leiden University, Leiden, Netherlands

**Keywords:** data mining, electronic medical record, psychiatric inpatient ward, seclusion, text mining

## Abstract

**Aim:** With the introduction of “Electronic Medical Record” (EMR) a wealth of digital data has become available. This provides a unique opportunity for exploring precedents for seclusion. This study explored the feasibility of text mining analysis in the EMR to eventually help reduce the use of seclusion in psychiatry.

**Methods:** The texts in notes and reports of the EMR during 5 years on an acute and non-acute psychiatric ward were analyzed using a text mining application. A period of 14 days was selected before seclusion or for non-secluded patients, before discharge. The resulting concepts were analyzed using chi-square tests to assess which concepts had a significant higher or lower frequency than expected in the “seclusion” and “non-seclusion” categories.

**Results:** Text mining led to an overview of 1,500 meaningful concepts. In the 14 day period prior to the event, 115 of these concepts had a significantly higher frequency in the seclusion category and 49 in the non-seclusion category. Analysis of the concepts from days 14 to 7 resulted in 54 concepts with a significantly higher frequency in the seclusion-category and 14 in the non-seclusion category.

**Conclusions:** The resulting significant concepts are comparable to reasons for seclusion in literature. These results are “proof of concept”. Analyzing text of reports in the EMR seems therefore promising as contribution to tools available for the prediction of seclusion. The next step is to build, train and test a model, before text mining can be part of an evidence-based clinical decision making tool.

## Introduction

Reasons for being admitted to a closed psychiatric ward usually involve the combination of psychiatric symptoms and aggressive or impulsive behaviors and/or presenting a risk to others or oneself (1–3). By providing structure, socio-therapeutic interventions, and medication, patients usually become less agitated (4, 5). In some situations, however, there is no other alternative than to use restraining measures (6). In the Netherlands, seclusion is the preferred restraining measure and is used more often compared to other countries, with forced medication being used less. The high use of seclusion (in number and duration) has been subject to national extensive political discussion and media coverage (7–9). Seclusion should be avoided as much as possible and not only because the therapeutic value is doubtful (10, 11). This measure has proven to be a traumatic intervention for both the patient (12, 13) and staff (14, 15). Various initiatives have taken place to diminish the use of seclusion (16–18). Over the past years seclusion rates in the Netherlands have lessened due to several reduction endeavors, such as the implementation of a High Intensive Care model in acute psychiatric wards (19–21). However, seclusion rates in the Netherlands still remain one of the highest compared to other countries. More efforts are needed to reduce the use of seclusion (8, 9).

Risk assessment has shown to be effective in reducing seclusion and is often incorporated in reduction efforts (8, 21–23). Reviews show a scarcity of well-designed studies addressing feasibility and effectiveness of de-escalating interventions as Gaynes et al. (24) remarked “The available evidence about relevant strategies is very limited. Only risk assessment decreased subsequent aggression or reduced use of seclusion and restraint (low strength of evidence). Evidence for de-escalating aggressive behavior is even more limited.”

The present article describes an innovative way of extracting words from the text available in the “Electronic Medical Record” (EMR) of patients admitted to psychiatric admission wards in order to predict seclusion (or assess risk); the focus here is on the prevention of seclusion as this is the most frequently used restraining measure in The Netherlands. The “Electronic Medical Record” (EMR) gives access to clinical data that was not readily available before its implementation. It allows large-scale clinical analysis in daily routines in psychiatry, however, the precise extraction of clinical relevant data from the narrative medical and nursing notes and other files can be challenging. An example of strategies used to extract data from texts is the study of Perlis et al. (25) who used “Natural Language Processing” for a chart review by processing text into meaningful concepts on a set of rules. They were able to give a proper indication of the patients that could be regarded to “become therapy resistant.” Cerrito et al. (26) wrote a white paper on the use of data-mining techniques on Electronic Medical Record in the emergency department of a hospital to improve care while lowering costs. They discovered that patients with similar complaints were treated very differently depending on the attending physician, and those differences can have an impact on both costs and care. Other examples are: predicting future risk of suicidal behavior using longitudinal historical data in electronic health records (27) or after discharge from general hospitals (28), detecting specific follow-up appointment criteria in hospital discharge records (29), extracting employment information of service members from the Electronic Health Record (30), identifying tapering patterns in switching of different antipsychotics (31) or identifying knowledge gaps in guidelines and exploring physicians' therapeutic decisions with data mining techniques to fill these knowledge gaps (32).

In the current explorative study text mining software is used to allow analysis of large amounts of text in which (patterns of) words are screened on whether or not they are more numerous in patients who are subsequently secluded. This method of analysis provides insight into what is relevant, what is related and what is representative from a large body of unstructured text (33). This technology has been used in several academic studies to perform text analysis in the medical domain (34, 35). The intention of this study is purely to explore the use of text mining in daily psychiatric practice to determine if it could be a viable tool in reducing the use of seclusion in the future. If the results are promising the next step would be to link qualitative information from the “Electronic Medical Record” (EMR) to a predictive model of seclusion. After validation, this model could provide the opportunity to develop a screening-algorithm that checks in “real time” if the relevant “trigger” (or “discriminative”) words and word-combinations (concepts) linked to seclusion appear in the “Electronic Medical Record” (EMR), thus giving a warning sign that a patient is at risk. This will provide means to de-escalate the behavior at an early stage and in turn reduce the number of seclusions. Such an alerting system should not lead to extra workload for the staff, be safe and have no negative impact on patient care and well-being.

The authors sought to answer the following question in this explorative study: could analyzing text in the files of patients be useful in the quest to reduce the use of seclusion in psychiatric practice? To answer this, the first step was to see if text mining in the Electronic Medical Record (EMR) could lead to the identification of meaningful concepts in the EMR that are numerically the most frequent in the medical files of the patients. The second step was to answer the question if any of these concepts typically relate to either a subsequent seclusion or, for non-seclusion, a subsequent discharge from the ward.

This study was purely explorative in nature to determine if text mining the EMR could result in useful concepts that typically precede seclusion on a psychiatric closed ward. This study is based on data mining: not hypothesis driven but data driven. The authors did not choose to formulate an expected outcome of concepts related to seclusion or non-seclusion. To the authors' knowledge, no studies were available at that time that indicated certain concepts would have a predictive value for seclusion or non-seclusion.

## Methods

### Study Design

A retrospective cohort study using unstructured data from routine patient reports and notes stored in the EMR written by nurses and physicians.

### Setting

The study took place in a large regional psychiatric hospital in The Netherlands with an urban catchment area of ~550,000 inhabitants. Data was gathered from an acute psychiatric admission ward which held 52 beds and 6 seclusion rooms (~1,300 patients admitted per annum on average with a mean length of stay of 16 days) and from a non-acute psychiatric admission ward with 42 beds and 2 seclusion rooms (around 300 patients admitted per annum on average with a mean length of stay of 42 days) (3).

### Participants

All nursing notes and medical reports written about patients admitted during the period August 2008–July 2012, on either the acute or the non-acute admission ward, were extracted from the EMR. Hence, including readmitted patients and secluded or non- secluded patients. Every note and report was used of every single patient to fully reflect day-to-day psychiatric practice, including possible missing information in the EMR.

### Procedure

After approval of the board of directors a request was made to the department of Internal Business Intelligence to extract all reports and notes from the EMR of the above described participants. These text files were deleted after the study and were anonymously analyzed by an external company which developed a text mining program.

### Analysis

**Figure 1:**
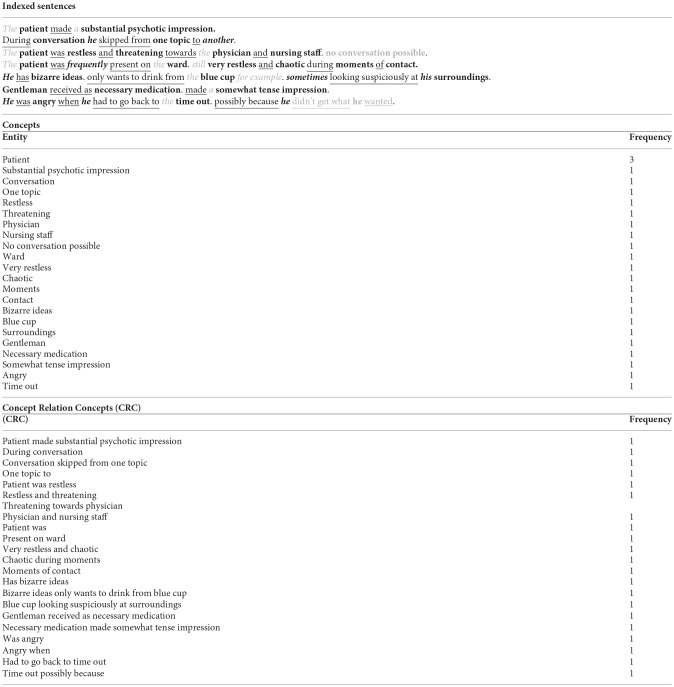
Example of text mining analysis. **Bold** are the concepts. underlined are the relationships. Grey and italic are unimportant words such as articles (‘the'. ‘a'). ***Black and italic*** are words that add to the concept such as pronouns or adverbs (‘he'. ‘sometimes'). Grey marking is a possible negation.

The goal of analysis was to first find frequently used concepts in the EMR and secondly if any of these concepts relate to either seclusion or non-seclusion of patients. Concepts were identified using text mining software. All the unstructured data in the EMR involving the day-to-day notes by the nursing staff and various psychiatric reports by physicians and other mental health professionals (excluding medication prescription) were analyzed using text mining software^1^. The approach of the software is to break texts into sentences, and to parse sentences into concepts and relation patterns, without predefined domain knowledge. The semantics analysis run by the software recognizes key elements such as concepts, relations, non-relevant words, and negations. Relations are commonly verbs, and nouns with adjusting words are concepts (Figure 1). The software itself automatically generates the most frequently used concepts. Frequency of concept is the number of times a concept appears in a text; note that this is not the same as the frequency of a word, because a concept can consist of multiple words (33).

The concepts of secluded patients were analyzed during a maximum of 2 weeks prior to seclusion and were compared to the concepts in reports of non-secluded patients during the last 14 days of their admission. To control for the differences in the time admitted in the hospital and differences between the acute and non-acute ward, a period of 14 days prior to seclusion vs. the last 14 days of admission for the non-secluded patients was selected for this study. The last 14 days of admission was chosen for the non-secluded group, because this is the most stable phase for them. These periods were not compared in the same time-frames. In this strategy there is no “control group” in a strict sense, but only a dichotomy: a patient is either secluded or not.

Chi-square analyses were used to test if there was a significant difference in the frequency of the concepts for the secluded and non-secluded categories during the 14 days prior to the event. Additionally, concepts from days 14 to 7 prior to either seclusion or discharge were analyzed in the same way. A Bonferroni correction was applied on the *p*-value to correct for the multiple hypothesis testing; i.e., 1,500 hypotheses, one for each concept, were tested.

### Ethical Considerations

Before conducting the study the authors consulted the Dutch Central Committee on Research Involving Human Subjects (CCMO) under the Dutch Medical Research Involving Human Subjects Act (WMO) regarding if approval of this study was needed. Seeing that this study does not include physically involved patients, interventions or subject patients to procedures that require them to follow rules of behavior, no approval of the ethical committee was sought. The study was approved by the medical director of the institute.

## Results

The study included 3,045 admissions for an acute psychiatric ward and a non-acute psychiatric ward from August 2008–July 2012. This accounted for 67,590 notes and reports of which 57,381 belonged to non-secluded patients and 10,209 to secluded patients. The total reports involved 2,816 patients of whom 1,687 (60%) were male and 1,129 (40%) were female. The mean age was 41 years (SD = 13) and 656 (23%) patients were secluded. The major diagnoses in this group were: schizophrenia (*N* = 967; 32%), mood disorders (*N* = 767; 25%), and other psychotic disorders (*N* = 672; 22%; Table 1).

**Table 1:** Demographic variables of the patients included in the three studies.

**DEMOGRAPHIC VARIABLE**	
Registrations (*N*)	3,045
Patients (*N*)	2,816
**GENDER** ***N*** **(%)**	
Male	1,687 (59.9%)
Female	1,129 (40.1%)
**AGE (YEARS)**	
Mean	41
SD	13
Min-Max	18-90
**SECLUDED** ***N*** **(%)**	
Yes	656 (23.3%)
No	2,160 (76.7%)
**DIAGNOSIS** ***N*** **(%)**	
Schizophrenia	967 (34%)
Mood disorders	767 (27%)
Psychotic disorders	672 (24%)
Alcohol dependence	360 (13%)
Drug dependence	265 (9%)
Adjustment disorders	238 (8%)
Anxiety/Somatoform/	
Dissociative disorders	188 (7%)
All other diagnoses	≤186 (7%)

*Diagnoses include all major diagnoses; patients typically have more than one major diagnosis in this population*.

The results were incorporated in a dashboard that computes graphs and tables when selecting a particular word or socio-demographic variable. Furthermore, the text mining analysis resulted in an overview of 1,500 (most meaningful) generated concepts from the EMR. The frequencies of these concepts were displayed for each of the 14 days prior to seclusion and discharge (non-seclusion). In total 1,500 concepts were mentioned 428,587 times, of which 67,088 were found in files of secluded patients and 361,499 in files of non-secluded patients. The overview of 1,500 concepts consisted of a number of repetitions that were seen as different concepts due to spelling or the use of abbreviations by staff. This was for example the case for the concepts regarding: mania, depression, hallucinations, paranoia, seclusion, and time-out room.

Chi-square analyses of all concepts and the occurrence of the concept in files of secluded or non-secluded patients in the 14 days prior to the event of seclusion or discharge, resulted in 115 concepts relating significantly to seclusion, ranging from the concept *seclusion* (Dutch abbreviation; χ^2^(1) = 287.89, *p* < 0.001) to the concept *fell down* (χ^2^(1) = 17.37, *p* < 0.05; Table 2). For the non-secluded patients significant relationships were found for 49 concepts, ranging from the concept *furlough* (χ^2^(1) = 238.34, *p* < 0.001) to the concept *sitting room* (χ^2^(1) = 18.17, *p* < 0.05; Table 3).

**Table 2:** Significant concepts for seclusion from notes and reports in the EMR (Chi-square).

	***N*** **(nsecl)**	***N*** **(secl)**	**% of concepts nsecl**	**% of concepts secl**	***N*** **(total)**	**% of all concepts**	**Exp*N*** **(nsecl)**	**Exp** ***N*(secl)**	**Chi-square**	**df**	*p*-value^*^
Seclusion (Dutch abbreviation)	165	163	0.00	0.00	328	0.00	276.66	51.34	287.89	1	0.00
Behavior	431	272	0.00	0.00	703	0.00	592.96	110.04	282.60	1	0.00
Threatening	29	56	0.00	0.00	85	0.00	71.69	13.31	162.43	1	0.00
Office	514	246	0.00	0.00	760	0.00	641.03	118.97	160.83	1	0.00
Time out room	32	57	0.00	0.00	89	0.00	75.07	13.93	157.85	1	0.00
Psychotic impression	93	87	0.00	0.00	180	0.00	151.82	28.18	145.60	1	0.00
t.o^**^	13	37	0.00	0.00	50	0.00	42.17	7.83	128.92	1	0.00
Psychotic	281	149	0.00	0.00	430	0.00	362.69	67.31	117.54	1	0.00
Time-out room	23	40	0.00	0.00	63	0.00	53.14	9.86	109.20	1	0.00
Very psychotic	30	42	0.00	0.00	72	0.00	60.73	11.27	99.34	1	0.00
Very restless	54	47	0.00	0.00	101	0.00	85.19	15.81	72.95	1	0.00
Agreements	403	162	0.00	0.00	565	0.00	476.56	88.44	72.54	1	0.00
Cigarettes	86	60	0.00	0.00	146	0.00	123.15	22.85	71.58	1	0.00
Door	523	194	0.00	0.00	717	0.00	604.77	112.23	70.62	1	0.00
Ground	176	90	0.00	0.00	266	0.00	224.36	41.64	66.60	1	0.00
Charged	37	37	0.00	0.00	74	0.00	62.42	11.58	66.12	1	0.00
Hour	317	133	0.00	0.00	450	0.00	379.56	70.44	65.87	1	0.00
Security	30	33	0.00	0.00	63	0.00	53.14	9.86	64.37	1	0.00
Paranoid	34	35	0.00	0.00	69	0.00	58.20	10.80	64.28	1	0.00
Smokers' requisites	68	50	0.00	0.00	118	0.00	99.53	18.47	63.81	1	0.00
Verbal	108	65	0.00	0.00	173	0.00	145.92	27.08	62.95	1	0.00
No signs	51	42	0.00	0.00	93	0.00	78.44	14.56	61.33	1	0.00
Angry	345	138	0.00	0.00	483	0.00	407.39	75.61	61.05	1	0.00
Hard	89	57	0.00	0.00	146	0.00	123.15	22.85	60.49	1	0.00
Florid psychotic	16	24	0.00	0.00	40	0.00	33.74	6.26	59.58	1	0.00
Shower	79	52	0.00	0.00	131	0.00	110.49	20.51	57.35	1	0.00
Radio	31	31	0.00	0.00	62	0.00	52.29	9.71	55.40	1	0.00
Restless	354	136	0.00	0.00	490	0.00	413.30	76.70	54.35	1	0.00
Garden	1,139	331	0.00	0.00	1,470	0.00	1,239.90	230.10	52.45	1	0.00
Emergency medication	11	19	0.00	0.00	30	0.00	25.30	4.70	51.66	1	0.00
Suspicious	286	115	0.00	0.00	401	0.00	338.23	62.77	51.53	1	0.00
Direct	248	104	0.00	0.00	352	0.00	296.90	55.10	51.45	1	0.00
Alarm	10	18	0.00	0.00	28	0.00	23.62	4.38	50.16	1	0.00
Time out	68	44	0.00	0.00	112	0.00	94.47	17.53	47.38	1	0.00
Lorazepam	297	115	0.00	0.00	412	0.00	347.51	64.49	46.90	1	0.00
Medication	3,084	753	0.01	0.01	3,837	0.01	3,236.38	600.62	45.84	1	0.00
Correction	31	28	0.00	0.00	59	0.00	49.76	9.24	45.20	1	0.00
Boundaries	91	51	0.00	0.00	142	0.00	119.77	22.23	44.16	1	0.00
Agitated	223	92	0.00	0.00	315	0.00	265.69	49.31	43.82	1	0.00
Beginning	380	135	0.00	0.00	515	0.00	434.39	80.61	43.50	1	0.00
Time-out	67	42	0.00	0.00	109	0.00	91.94	17.06	43.21	1	0.00
Paranoid impression	54	37	0.00	0.00	91	0.00	76.76	14.24	43.10	1	0.00
Weed	26	25	0.00	0.00	51	0.00	43.02	7.98	43.00	1	0.00
God	43	32	0.00	0.00	75	0.00	63.26	11.74	41.45	1	0.00
Oxa	62	39	0.00	0.00	101	0.00	85.19	15.81	40.33	1	0.00
Demanding	42	31	0.00	0.00	73	0.00	61.57	11.43	39.75	1	0.00
Not sick	64	39	0.00	0.00	103	0.00	86.88	16.12	38.48	1	0.00
Hand	243	94	0.00	0.00	337	0.00	284.25	52.75	38.24	1	0.00
Force majeure	12	16	0.00	0.00	28	0.00	23.62	4.38	36.51	1	0.00
Agitation	97	49	0.00	0.00	146	0.00	123.15	22.85	35.46	1	0.00
Doors	44	30	0.00	0.00	74	0.00	62.42	11.58	34.71	1	0.00
Colleague	689	203	0.00	0.00	892	0.00	752.37	139.63	34.10	1	0.00
Closet	40	28	0.00	0.00	68	0.00	57.36	10.64	33.55	1	0.00
Directive	28	23	0.00	0.00	51	0.00	43.02	7.98	33.49	1	0.00
Night	706	206	0.00	0.00	912	0.00	769.24	142.76	33.22	1	0.00
Custody measure	442	142	0.00	0.00	584	0.00	492.58	91.42	33.19	1	0.00
Gone	699	204	0.00	0.00	903	0.00	761.65	141.35	32.92	1	0.00
1 h	47	30	0.00	0.00	77	0.00	64.95	12.05	31.68	1	0.00
Everyone	242	89	0.00	0.00	331	0.00	279.19	51.81	31.64	1	0.00
Confiscate	37	26	0.00	0.00	63	0.00	53.14	9.86	31.31	1	0.00
Psychotic utterances	77	40	0.00	0.00	117	0.00	98.69	18.31	30.44	1	0.00
Pointed	123	54	0.00	0.00	177	0.00	149.29	27.71	29.58	1	0.00
Water	92	44	0.00	0.00	136	0.00	114.71	21.29	28.73	1	0.00
Affectless impression	32	23	0.00	0.00	55	0.00	46.39	8.61	28.52	1	0.00
Window	65	35	0.00	0.00	100	0.00	84.35	15.65	28.35	1	0.00
Cigarette	123	53	0.00	0.00	176	0.00	148.45	27.55	27.87	1	0.00
Considerable	19	17	0.00	0.00	36	0.00	30.36	5.64	27.17	1	0.00
Affectless	111	49	0.00	0.00	160	0.00	134.95	25.05	27.16	1	0.00
Sleep	118	51	0.00	0.00	169	0.00	142.55	26.45	27.00	1	0.00
Very suspicious	39	25	0.00	0.00	64	0.00	53.98	10.02	26.56	1	0.00
Police	360	115	0.00	0.00	475	0.00	400.65	74.35	26.34	1	0.00
Claiming	104	46	0.00	0.00	150	0.00	126.52	23.48	25.61	1	0.00
Several times	54	30	0.00	0.00	84	0.00	70.85	13.15	25.60	1	0.00
Fluctuating	264	90	0.00	0.00	354	0.00	298.59	55.41	25.60	1	0.00
Very angry	72	36	0.00	0.00	108	0.00	91.09	16.91	25.57	1	0.00
Pounding	18	16	0.00	0.00	34	0.00	28.68	5.32	25.40	1	0.00
Eyes	153	60	0.00	0.00	213	0.00	179.66	33.34	25.27	1	0.00
Warning	30	21	0.00	0.00	51	0.00	43.02	7.98	25.16	1	0.00
Restless/boisterous presence	52	29	0.00	0.00	81	0.00	68.32	12.68	24.91	1	0.00
Mania	67	34	0.00	0.00	101	0.00	85.19	15.81	24.81	1	0.00
Incident	61	32	0.00	0.00	93	0.00	78.44	14.56	24.78	1	0.00
Mr. Last night	44	26	0.00	0.00	70	0.00	59.04	10.96	24.48	1	0.00
Mobile	47	27	0.00	0.00	74	0.00	62.42	11.58	24.33	1	0.00
Pills	62	32	0.00	0.00	94	0.00	79.29	14.71	24.08	1	0.00
5 o'clock	34	22	0.00	0.00	56	0.00	47.23	8.77	23.69	1	0.00
Seclusion	4	8	0.00	0.00	12	0.00	10.12	1.88	23.65	1	0.00
Cannabis	48	27	0.00	0.00	75	0.00	63.26	11.74	23.52	1	0.00
Tranxene	66	33	0.00	0.00	99	0.00	83.50	15.50	23.44	1	0.00
Complaint	37	23	0.00	0.00	60	0.00	50.61	9.39	23.38	1	0.00
Restless/boisterous	506	147	0.00	0.00	653	0.00	550.78	102.22	23.26	1	0.00
Naked	13	13	0.00	0.00	26	0.00	21.93	4.07	23.23	1	0.00
Question	440	131	0.00	0.00	571	0.00	481.62	89.38	22.98	1	0.00
Cooperative	211	74	0.00	0.00	285	0.00	240.39	44.61	22.95	1	0.00
Chaotic	148	57	0.00	0.00	205	0.00	172.91	32.09	22.93	1	0.00
Excuses	113	47	0.00	0.00	160	0.00	134.95	25.05	22.82	1	0.00
Restlessness	193	69	0.00	0.00	262	0.00	220.99	41.01	22.65	1	0.00
Bathroom	47	26	0.00	0.00	73	0.00	61.57	11.43	22.03	1	0.00
Uninhibited	62	31	0.00	0.00	93	0.00	78.44	14.56	22.02	1	0.00
Very restless/boisterous	95	41	0.00	0.00	136	0.00	114.71	21.29	21.64	1	0.00
Own room	117	47	0.00	0.00	164	0.00	138.33	25.67	21.01	1	0.01
Desperate	43	24	0.00	0.00	67	0.00	56.51	10.49	20.64	1	0.01
Trousers	32	20	0.00	0.00	52	0.00	43.86	8.14	20.49	1	0.01
Confused	81	36	0.00	0.00	117	0.00	98.69	18.31	20.25	1	0.01
Motoric	35	21	0.00	0.00	56	0.00	47.23	8.77	20.24	1	0.01
Forceful	149	55	0.00	0.00	204	0.00	172.07	31.93	19.76	1	0.01
Hands	157	57	0.00	0.00	214	0.00	180.50	33.50	19.55	1	0.01
Wall	57	28	0.00	0.00	85	0.00	71.69	13.31	19.24	1	0.02
Seclusion room	4	7	0.00	0.00	11	0.00	9.28	1.72	19.18	1	0.02
Substantial	64	30	0.00	0.00	94	0.00	79.29	14.71	18.83	1	0.02
Defensive	160	57	0.00	0.00	217	0.00	183.03	33.97	18.52	1	0.03
Nursing staff	1,111	275	0.00	0.00	1,386	0.00	1,169.05	216.95	18.41	1	0.03
Difficult	827	213	0.00	0.00	1,040	0.00	877.21	162.79	18.36	1	0.03
Physicians	47	24	0.00	0.00	71	0.00	59.89	11.11	17.71	1	0.04
Smoking area	190	64	0.00	0.00	254	0.00	214.24	39.76	17.52	1	0.04
Fell down	175	60	0.00	0.00	235	0.00	198.21	36.79	17.37	1	0.05

*N concepts seclusion = 67,088; N concepts non-seclusion = 361,499*.

*secl, secluded patients; nsecl, non-secluded patients*;

* *After Bonferroni correction*;

** *Time-out*.

*The original Dutch resulting concepts are included in the Supplementary Material (Data Sheet 1)*.

**Table 3:** Significant concepts for non-seclusion from notes and reports in the EMR (Chi-square).

	***N*(nsecl)**	***N*(secl)**	**% of concepts nsecl**	**% of concepts secl**	***N*(total)**	**% of all concepts**	**Exp** *N* **(nsecl)**	**Exp** *N* **(secl)**	**Chi-square**	**df**	*p*-value^*^
Furlough	2,264	97	0.01	0.00	2,361	0.01	1,991.43	369.57	238.34	1	0.00
Liberties	4,980	454	0.01	0.01	5,434	0.01	4,583.40	850.60	219.24	1	0.00
Friendly	7,296	789	0.02	0.01	8,085	0.02	6,819.43	1,265.57	212.76	1	0.00
Mr not awake	3,534	335	0.01	0.00	3,869	0.01	3,263.37	605.63	143.37	1	0.00
Ms not awake	1,492	83	0.00	0.00	1,575	0.00	1,328.46	246.54	128.61	1	0.00
Friendly present	1,978	149	0.01	0.00	2,127	0.00	1,794.05	332.95	120.49	1	0.00
Quiet	4,647	544	0.01	0.01	5,191	0.01	4,378.44	812.56	105.24	1	0.00
Good	6,914	894	0.02	0.01	7,808	0.02	6,585.79	1,222.21	104.49	1	0.00
Unnoticeably present	1,215	84	0.00	0.00	1,299	0.00	1,095.66	203.34	83.04	1	0.00
Not awake	1,597	144	0.00	0.00	1,741	0.00	1,468.48	272.52	71.86	1	0.00
Tomorrow	2,308	254	0.01	0.00	2,562	0.01	2,160.96	401.04	63.92	1	0.00
No symptoms	536	22	0.00	0.00	558	0.00	470.65	87.35	57.96	1	0.00
Home	866	63	0.00	0.00	929	0.00	783.58	145.42	55.38	1	0.00
All night not awake	572	34	0.00	0.00	606	0.00	511.14	94.86	46.29	1	0.00
Day structure	2,756	349	0.01	0.01	3,105	0.01	2,618.97	486.03	45.81	1	0.00
Helpful	501	27	0.00	0.00	528	0.00	445.35	82.65	44.42	1	0.00
Adequate	1,173	117	0.00	0.00	1,290	0.00	1,088.07	201.93	42.35	1	0.00
Impression	1,447	157	0.00	0.00	1,604	0.00	1,352.92	251.08	41.79	1	0.00
Whole night not awake	430	22	0.00	0.00	452	0.00	381.25	70.75	39.83	1	0.00
As usual	503	31	0.00	0.00	534	0.00	450.41	83.59	39.23	1	0.00
Contact	5,974	889	0.02	0.01	6,863	0.02	5,788.71	1,074.29	37.89	1	0.00
Return	550	38	0.00	0.00	588	0.00	495.96	92.04	37.62	1	0.00
Madam not awake	698	57	0.00	0.00	755	0.00	636.82	118.18	37.55	1	0.00
Group	1,488	172	0.00	0.00	1,660	0.00	1,400.16	259.84	35.21	1	0.00
Happy	623	51	0.00	0.00	674	0.00	568.50	105.50	33.38	1	0.00
House	2,252	294	0.01	0.00	2,546	0.01	2,147.47	398.53	32.51	1	0.00
Quietly present	2,272	299	0.01	0.00	2,571	0.01	2,168.55	402.45	31.52	1	0.00
Discharge	1,635	203	0.00	0.00	1,838	0.00	1,550.29	287.71	29.57	1	0.00
Present	2,866	402	0.01	0.01	3,268	0.01	2,756.45	511.55	27.81	1	0.00
Adequate impression	303	16	0.00	0.00	319	0.00	269.07	49.93	27.34	1	0.00
Unnoticeable	284	14	0.00	0.00	298	0.00	251.35	46.65	27.09	1	0.00
Somber	788	82	0.00	0.00	870	0.00	733.82	136.18	25.56	1	0.00
Slept	1,729	228	0.00	0.00	1,957	0.00	1,650.66	306.34	23.75	1	0.00
No psychotic utterances	345	25	0.00	0.00	370	0.00	312.08	57.92	22.18	1	0.00
Weekend	589	58	0.00	0.00	647	0.00	545.72	101.28	21.92	1	0.00
Whole night	882	101	0.00	0.00	983	0.00	829.13	153.87	21.54	1	0.01
ms m.i^**^	114	0	0.00	0.00	114	0.00	96.16	17.84	21.16	1	0.01
Contacts	312	22	0.00	0.00	334	0.00	281.72	52.28	20.79	1	0.01
Work	358	28	0.00	0.00	386	0.00	325.58	60.42	20.63	1	0.01
No characteristics	191	8	0.00	0.00	199	0.00	167.85	31.15	20.40	1	0.01
Admission	910	107	0.00	0.00	1,017	0.00	857.81	159.19	20.29	1	0.01
Suicidal tendencies	243	14	0.00	0.00	257	0.00	216.77	40.23	20.27	1	0.01
Manic state	180	7	0.00	0.00	187	0.00	157.73	29.27	20.09	1	0.01
Depressive state	250	15	0.00	0.00	265	0.00	223.52	41.48	20.04	1	0.01
No psychotic characteristics	262	17	0.00	0.00	279	0.00	235.33	43.67	19.31	1	0.02
Woman	530	53	0.00	0.00	58	0.00	491.74	91.26	19.02	1	0.02
Sport	631	68	0.00	0.00	699	0.00	589.58	109.42	18.59	1	0.02
Own way	642	70	0.00	0.00	712	0.00	600.55	111.45	18.28	1	0.03
Sitting room	2,809	417	0.01	0.01	3,226	0.01	2,721.02	504.98	18.17	1	0.03

*N concepts seclusion = 67.088; N concepts non-seclusion = 361.499*.

*secl, secluded patients; nsecl, non-secluded patients*;

* *After Bonferroni correction*;

** *m.i., medication intake*.

*The original Dutch resulting concepts are included in the Supplementary Material (Data Sheet 1)*.

Analysis of the concepts from days 14 to 7 involved 1,499 concepts (*letter of discharge* not yet mentioned in the reports and notes), which were mentioned in total 209,796 times in the EMR: 31,143 times in files of secluded patients and 178,653 times in files of non-secluded patients. Chi-square analyses led to 54 significant relating concepts to seclusion, ranging from the concept *behavior* (χ^2^(1) = 114.18, *p* < 0.001) to *not clear* (χ^2^(1) = 17.39, *p* < 0.05; Table 4). Compared to the full 14 days leading up to the event of seclusion, the following 68 concepts are not yet significant: *mania, 5 o'clock, several times, paranoid impression, defensive, agitation, physicians, bathroom, pounding, angry, trousers, cannabis, chaotic, claiming, colleague, cooperative, doors, directive, restless/boisterous, restless/boisterous presence, forceful, demanding, very suspicious, very restless/boisterous, very psychotic, excuses, substantial, fell down, god, boundaries, ground, hand, hands, custody measure, everyone, closet, complaint, lorazepam, mobile, difficult, motoric, wall, naked, night, eyes, affectless, affectless impression, restlessness, restless, uninhibited, force majeure, pills, psychotic utterances, smoking area, seclusion, seclusion room, cigarette, sleep, tranxene, verbal, nursing staff, confused, question, warning, desperate, water, gone*, and *fluctuating*. In this week before the event of seclusion, seven additional concepts were significant but were not significant in the full 14 days before seclusion. These are the concepts: *ambulant practitioner, short, loud, not clear, schedule, hunch/suspicion*, and *early shift*.

**Table 4:** Signicifant concepts 7 days prior to seclusion.

**Concept**	**Higher frequency in category**	**Chi-square**	**df**	*p*-value^*^
Behavior	Seclusion	114.18	1	0.00
Office	Seclusion	106.85	1	0.00
Psychotic impression	Seclusion	85.83	1	0.00
Seclusion	Seclusion	85.76	1	0.00
Smokers' requisites	Seclusion	74.12	1	0.00
Charged	Seclusion	68.62	1	0.00
t.o^**^	Seclusion	67.17	1	0.00
No signs	Seclusion	65.10	1	0.00
Threatening	Seclusion	60.84	1	0.00
Agreements	Seclusion	56.58	1	0.00
Alarm	Seclusion	45.80	1	0.00
Correction	Seclusion	44.85	1	0.00
Beginning	Seclusion	44.11	1	0.00
Time out room	Seclusion	43.07	1	0.00
Time-out	Seclusion	43.02	1	0.00
Cigarettes	Seclusion	42.83	1	0.00
Hour	Seclusion	42.18	1	0.00
Window	Seclusion	41.15	1	0.00
Very restless	Seclusion	41.06	1	0.00
Time-out room	Seclusion	40.33	1	0.00
Garden	Seclusion	39.96	1	0.00
Time out	Seclusion	39.86	1	0.00
Very angry	Seclusion	39.34	1	0.00
Oxa	Seclusion	37.99	1	0.00
Door	Seclusion	37.44	1	0.00
Mr last night	Seclusion	37.35	1	0.00
Ambulant practitioner	Seclusion	37.31	1	0.00
Not sick	Seclusion	37.24	1	0.00
Own room	Seclusion	36.15	1	0.00
Emergency medication	Seclusion	33.44	1	0.00
1 h	Seclusion	31.19	1	0.00
Psychotic	Seclusion	29.47	1	0.00
Hard	Seclusion	29.09	1	0.00
Short	Seclusion	28.97	1	0.00
Hunch/suspicion	Seclusion	28.75	1	0.00
Weed	Seclusion	28.75	1	0.00
Medication	Seclusion	27.79	1	0.00
Pointed	Seclusion	27.32	1	0.00
Early shift	Seclusion	24.03	1	0.00
Direct	Seclusion	23.64	1	0.00
Agitated	Seclusion	23.53	1	0.00
Shower	Seclusion	23.30	1	0.00
Suspicious	Seclusion	22.13	1	0.00
Considerable	Seclusion	21.70	1	0.00
Radio	Seclusion	20.62	1	0.01
Confiscate	Seclusion	20.16	1	0.01
Loud	Seclusion	19.86	1	0.01
Security	Seclusion	19.52	1	0.01
Florid psychotic	Seclusion	19.52	1	0.01
Incident	Seclusion	18.72	1	0.02
Paranoid	Seclusion	18.71	1	0.02
Police	Seclusion	17.75	1	0.04
Schedule	Seclusion	17.52	1	0.04
Not clear	Seclusion	17.39	1	0.05

* *After Bonferroni correction*;

** *time-out*.

*The original Dutch resulting concepts are included in the Supplementary Material (Data Sheet 1)*.

Regarding concepts relating to non-seclusion, days 14 to 7 were significant during days 14 to 7 prior to discharge. These comprised of 35 less concepts that were significant than in the analysis of the full 14 days (Table 5). Concepts that were no longer significant were the following: *depressive state, present, adequate, adequate impression, helpful, happy, contacts, day structure, own way, as usual, no characteristics, no psychotic characteristics, no psychotic utterances, whole night, all night not awake, group, house, sitting room, impression, manic state, madam not awake, tomorrow, ms m.i, unnoticeable, discharge, admission, return, quietly present, slept, somber, sport, suicidal tendencies, woman, weekend*, and *work*.

**Table 5:** Signicifant concepts 7 days prior to discharge (non-seclusion).

**Concept**	**Higher frequency in category**	**Chi-square**	**df**	*p*-value^*^
Liberties	Non-seclusion	88.99	1	0.00
Friendly	Non-seclusion	72.06	1	0.00
Furlough	Non-seclusion	67.53	1	0.00
Good	Non-seclusion	62.44	1	0.00
Mr not awake	Non-seclusion	51.57	1	0.00	
Ms not awake	Non-seclusion	50.43	1	0.00	
Friendly present	Non-seclusion	49.57	1	0.00	
Home	Non-seclusion	41.32	1	0.00
Quiet	Non-seclusion	41.09	1	0.00
Not awake	Non-seclusion	27.65	1	0.00	
Unnoticeably present	Non-seclusion	22.85	1	0.00	
Contact	Non-seclusion	21.05	1	0.01
No symptoms	Non-seclusion	17.99	1	0.03	
Whole night not awake	Non-seclusion	17.36	1	0.05	

* *After Bonferroni correction*.

*The original Dutch resulting concepts are included in the Supplementary Material (Data Sheet 1)*.

## Discussion

The present study explored the usefulness of analyzing text in the files of patients to identify concepts from reports and notes written by nurses and physicians that typically precede the incidence of seclusion. The authors were looking for a “proof of concept.” Would it be possible to differentiate or identify concepts that precede seclusion? Text mining led to a list of 1,500 meaningful concepts from the EMR that are numerical the most frequent in files of patients. Of these 1,500 concepts, 115 seem to typically precede seclusion during 14 days. At first glance the majority of these 115 concepts correspond to (intuitive) clinical experience and can be viewed as five groups:

1. phrases that accompany reasons to use seclusion (i.e., concepts comprising the phrases: threatening, psychotic, restlessness, paranoia, verbal, angry, agitated, affectless, claiming, pounding, mania, chaotic, uninhibited, confusion, and custody measure). These phrases are in line with literature that describe the reasons for using seclusion or restraint in psychiatric inpatient practice. For instance Keski-Valkama et al. (36) found that agitation/disorientation was the most frequent reason for the use of restraint and seclusion. Knutzen et al. (37) discovered that the restrained group in their study consisted of a large proportion of psychosis related primary diagnoses. Larue et al. (38) describe that the main reasons for seclusion were agitation, disorganization and aggressive behavior. Vollema et al. (39) found that the risk for seclusion increases in the presence of irritable/aggressive behavior, motoric restlessness, and the decrease of the feeling of safety among staff. Bowers et al. (40) mention aggressive behavior as a reason for seclusion. El-Badri and Mellsop (41) found that a primary diagnosis of schizophrenia, mania and substance abuse tended to be secluded more frequently than others and also threats of violence to staff, property and actual violence. Husum et al. (42) discovered that patients who are overactive and aggressive, experiencing hallucinations and delusions, executing self-injury or at risk of suicide have a higher risk of being secluded and restrained than patients not showing such behavior. They also found that diagnosis of schizophrenia or other psychosis was linked to seclusion. Tunde (43) wrote that those that were secluded were more likely to be young, involuntarily admitted, had a diagnosis of schizophrenia, were a risk to others, risk to self and at risk of absconding. Noorthoorn et al. (9) reported that higher seclusion rates were associated with psychotic disorders and male gender.
2. Other containment measures used in psychiatric practice (i.e., the concepts including time out and emergency medication). These “alternative” containment measures are for example described by Dack et al. (44). They defined a number of containment measures used in psychiatric practice, such as seclusion, PRN medication, physical restraint, time out, compulsory intramuscular medication.
3. implementing seclusion (i.e., the concepts: seclusion (three concepts—different spelling or abbreviation), ground, security, alarm, force majeure, and police). These concepts seem to describe the process of secluding a patient.
4. the working environment of nursing staff. For example the concepts: office, medication, colleague, confiscate, and physicians.
5. non-specific terms, such as cigarette, radio, night, everyone, water, bathroom, and 5 o'clock.

The concepts that show a relationship with non-seclusion also have face validity and seem to describe unobtrusive and calm patients. Striking are the words relating to depression and suicidal behavior. This does not seem to resonate with, for example, one of the findings of Vollema et al. (39) that depression was more common among those who were secluded. Also the word woman seems to be in line with El-Badri et al.'s (41) finding that men were more likely than women to be secluded.

It was interesting to look at the significant relationships of the concepts a week before the event of seclusion or discharge. A little more than half of the concepts that were significant in the full 14 days were significant during the days 14 to 7. Even though a lot of the words are not yet significant, there are still words that describe reasons for seclusion (i.e., agitated, charged, threatening, psychosis) and the use of other containment measures (i.e., time out and emergency medication). This could mean that a seclusion can be predicted a week before commencing and makes text mining an interesting tool in the quest of reducing the use of seclusion. However, about one third of secluded patients are secluded more than once during an admission and seclusion usually takes place in the first week of admission (41, 43). This could be a confounding factor in the concepts found in this study, as some are already describing a seclusion incident.

This study took place during nationwide seclusion reduction initiatives that also affected the culture on most admission wards in The Netherlands (10, 14) and resulted in a reduction of seclusion rates (10–12). These changes are not expected to have an impact on the presently found results and conclusions. The reason is that text-mining reflects the culture and way of working on a specific ward. Regarding concepts related to seclusion that describe the reason for using the restraining measure: these are expected to result in similar words, as reasons for using a restraining measure are universal (usually relating to aggression).

There are several limitations to this study. The present study used a particular text mining application. There are several other applications for text mining available on the market, which analyze text in the same way. Perhaps if the present study used different software the results would be different. This, however, is not to be expected.

A limitation is the question of generalizability. This study was conducted on a specific ward in the Netherlands, using Dutch words which may translate differently in other languages. Nevertheless, using text mining in a particular ward always starts with a baseline and training a model in the particular setting. It could be quite possible that depending on cultural or clinical setting and language other concepts can be identified in the EMR that precede or predict seclusion. However, it does seem plausible that similar concepts as found here will also result on another closed psychiatric ward (with the exception of phrases used in a particular hospital, such as the name of the ward or codes used to describe symptoms), because similar phrases as reasons for seclusion are also described in literature. But it is important to keep in mind that the present results only give an indication that text mining the EMR in this context is feasible. Another limitation is that staff do not report in the same way, such as using abbreviations or another spelling for words. The same word can be noted differently in the EMR. For example time-out room: t.o., time-out room, time out room, time-out room. The software did not seem to include these as same entities and resulted in these concepts having a lower frequency. These concepts will therefore have to be manually identified in the exploration phase and combined as input for a possible future predictive model. However, taking into consideration that this study was conducted several years ago and the field of data analysis has evolved and is momentarily thriving, it could be expected that these duplicates of concepts would already be considerably diminished in the first step of analysis with present day updated and new software. Furthermore, the period of 14 days studied here was not compared for the individual patients in the same time-frames. There could be confounding factors involved in these different timeframes, such as an incident that has taken place on the ward or the time of the year. Also, it could be that some staff members view certain patients in a biased way and write their reports accordingly. Additionally, each of the 14 days may not comprise of a comparable quantity of reports that were analyzed. It is advised that future analysis controls for this by making “buckets” of reports to improve comparison. Also, perhaps non-secluded patients as a comparison group can be selected in the middle of admission and not before discharge. This could possibly lead to less discharge-related concepts.

The most important future direction is building and testing a predictive model, for example as described in Barak-Corren et al. (27). In the future perhaps a trained and tested text mining model could lead to “real time” analysis of all day-to-day notes and reports in the Electronic Medical Record. This means that the staff can continue their “routine” way of recording without increasing administrative workload and in the meantime be supported in their judgment and prediction about patients at risk for seclusion. This judgement could be based, for example, on routinely applied structured risk assessment scales (Crisis Monitor) (22). With the use of a specific “User Interface,” data derived from the EMR database can be “transformed” into real time risk assessing information, indicating the probability of seclusion. This can, through a predictive algorithm, yield the signals per individual patient, for example: green indicating no problem, orange indicating providing extra preventative care for the patient and red indicating immediate action needed. Either at the nursing station or on a handheld device, a warning can be generated per individual patient. The type and sequence of the interventions in phase orange or red can be protocolled both in a general way and tailored to specific patient needs. On the basis of continuous feedback, validity of the system can be upgraded and adapted. Ultimately it can be fine-tuned to local resources and attitudes leading to a Clinical Decision Support System. This enhances safety of patients and staff in general, not only with regard to seclusion. Another aspect is that it may also support inter staff communication on a continuous base in an effective and efficient adjuvant way.

It is clear that this approach can also be used in many other contexts. Currently our institution is looking into the possibilities of text mining to support Assertive Community Teams with this approach to diminish (involuntary) admissions and screen outpatients for suicidal tendencies.

Altogether, these results answer the research question positively and it seems to be feasible to identify certain concepts in the EMR that typically precede a seclusion episode. These premature findings may be regarded as a “proof of concept” to use text in the EMR from patients admitted to an (acute) admission ward to help predict subsequent seclusion. Furthermore, these results may help process implicit (clinical) knowledge to become formal knowledge. As mentioned before, this is a pure exploratory study and the study should be repeated, a model built, trained and tested and further evaluation and validation before becoming part of an evidence-based clinical decision making tool. However, the results seem promising that “real time” text analysis of the EMR may be a clinical feasible and possible efficient way to identify patients at risk for seclusion in the future. Thus, offering opportunities for less invasive alternative interventions.

## Author Contributions

EH initiated the idea to use text mining techniques on reports in the EMR to predict seclusion. EH initiated the collaboration with Intersystems. RdW made it possible to gather data from the specific wards. MH and NM were responsible for delivering the EMR data for text mining analysis. DvH and DW used their company's software to analyze the data. MH wrote the article together with RdW and EH. RvE was responsible for the chi-square analyses.

### Conflict of Interest Statement

DvH and DW were employees for Intersystems BV Benelux at the time of the study. The remaining authors declare that the research was conducted in the absence of any commercial or financial relationships that could be construed as a potential conflict of interest.

## Footnotes

^1^iKnow smart indexing©, Intersystems.
