# Supplementary material for: Text Analysis of Electronic Medical Records to Predict Seclusion in Psychiatric Wards: Proof of Concept
Source: Front Psychiatry. 2019 Apr 11;10:188. doi: 10.3389/fpsyt.2019.00188 (PMC6470375; doi:10.3389/fpsyt.2019.00188)
Supplement: Supplementary file 1 [file Data_Sheet_1.docx]

**Appendix 1. Concepts tables 2 through 5 in Dutch**

**Table 2. Table 2 (*continued*) Table 3.**

separeer

gedrag

dreigend

kantoor

time out kamer

psychotische indruk

t.o

psychotisch

time-out kamer

erg psychotisch

erg onrustig

afspraken

sigaretten

deur

grond

geladen

uur

beveiliging

paranoide

rookwaar

verbaal

geen tekenen

boos

hard

floride psychotisch

douche

radio

onrustig

tuin

noodmedicatie

achterdochtig

direct

alarm

time out

lorazepam

medicatie

correctie

grenzen

geagiteerd

begin

time-out

achterdochtige indruk

wiet

god

oxa

eisend

niet ziek

hand

overmacht

agitatie

deuren

collega

kast

directief

nacht

ibs

weg

1 uur

iedereen

beslag

psychotische uitspraken

gewezen

water

oninvoelbare indruk

raam

sigaret

fors

oninvoelbaar

slaap

erg achterdochtig

politie

claimend

aantal keer

wisselend

erg boos

bonken

ogen

waarschuwing

druk aanwezig

2a manie

incident

dhr vannacht

mobiel

pillen

5 uur

separatie

cannabis

tranxene

klacht

druk

naakt

vraag

cooperatief

chaotisch

excuses

onrust

badkamer

ontremd

erg druk

eigen kamer

wanhopig

broek

verward

motorisch

dwingend

handen

muur

separeerruimte

flink

afwerend

verpleging

moeilijk

artsen

rookruimte

gevallen

verlof

vrijheden

vriendelijk

dhr niet wakker

mw niet wakker

vriendelijk aanwezig

rustig

goed

onopvallend aanwezig

niet wakker

morgen

geen doelsymptomen

thuis

hele nacht niet wakker

dagstructuur

behulpzaam

adequaat

indruk

gehele nacht niet wakker

gangetje

contact

retour

mevr niet wakker

groep

blij

huis

rustig aanwezig

ontslag

aanwezig

adequate indruk

onopvallend

somber

sliep

geen psychotische uitspraken

weekend

gehele nacht

mw m.i

contacten

werk

geen kenmerken

opname

suicidaliteit

manische stemming

2b depressieve stemming

geen psychotische kenmerken

vrouw

sport

eigen gang

huiskamer

**Table 4. Table 5.**

gedrag

kantoor

psychotische indruk

separeer

rookwaar

geladen

t.o

geen tekenen

dreigend

afspraken

alarm

correctie

begin

time out kamer

time-out

sigaretten

uur

raam

erg onrustig

time-out kamer

tuin

time out

erg boos

oxa

deur

dhr vannacht

ambulant behandelaar

niet ziek

eigen kamer

noodmedicatie

1 uur

psychotisch

hard

kort

vermoeden

wiet

medicatie

gewezen

vroege dienst

direct

geagiteerd

douche

achterdochtig

fors

radio

beslag

luidruchtig

beveiliging

floride psychotisch

incident

paranoide

politie

schema

niet duidelijk

vrijheden

vriendelijk

verlof

goed

dhr niet wakker

mw niet wakker

vriendelijk aanwezig

thuis

rustig

niet wakker

onopvallend aanwezig

contact

geen doelsymptomen

hele nacht niet wakker
